# Functionalized Cellulose Networks for Efficient Oil Removal from Oil–Water Emulsions

**DOI:** 10.3390/polym8020052

**Published:** 2016-02-17

**Authors:** Uttam C. Paul, Despina Fragouli, Ilker S. Bayer, Athanassia Athanassiou

**Affiliations:** Smart Materials, Nanophysics, Istituto Italiano di Tecnologia, via Morego 30, 16163 Genova, Italy; uttam.paul@iit.it (U.C.P.); ilker.bayer@iit.it (I.S.B.); athanassia.athanassiou@iit.it (A.A.)

**Keywords:** cellulose, emulsions, oil–water separation, hydrophilicity, underwater superoleophobicity

## Abstract

The separation of oil from water in emulsions is a great environmental challenge, since oily wastewater is industrially produced. Here, we demonstrate a highly efficient method to separate oil from water in non-stabilized emulsions, using functionalized cellulose fiber networks. This is achieved by the modification of the wetting properties of the fibers, transforming them from oil- and water-absorbing to water-absorbing and oil-proof. In particular, two diverse layers of polymeric coatings, paraffin wax and poly(dimethylsiloxane)-*b*-poly(ethylene oxide) (PDMS-*b*-PEO) diblock copolymer, are applied on the surface of each individual fiber by a two-step dip adsorption process. The resulting cellulose networks exhibit superhydrophilicity and underwater superoleophobicity and they are mechanically reinforced. Therefore, the described treatment makes cellulose fiber networks excellent candidates for the filtration and subsequent removal of oil from oil-in-water non-stabilized emulsions with oil separation efficiency up to 99%. The good selectivity, reproducibility, and cost effectiveness of the preparation process leads to the production of low cost filters that can be used in oil–water separation applications.

## 1. Introduction

Owing to the large quantities of industrial emulsified wastewater from petrochemical, chemical, and mineral industries, as well as frequent oil spill accidents, oil–water separation is an important ecological problem [1,2]. As a result, a great deal of effort has been dedicated to the surface modification of polymeric woven or non-woven porous materials, turning them into systems able to efficiently separate oil from water. Such materials present advantages due to the large surface-to-volume ratio, but also due to the synergistic effect of the properties of the individual components such as the mechanical properties of the polymeric support and the functionalities of the developed organic or inorganic layers. In particular, hydrophobic-oleophilic or hydrophilic-oleophobic functionalities are introduced on filter paper (FP), polymer films, foams, and textiles [2,3,4,5,6,7,8,9,10,11,12,13,14,15] upon the utilization of macromolecular layers, nano or micro-particles, nanofibers, *etc.*, making these materials able to separate floating oils on water or water–oil emulsions.

Cellulose networks are porous, micro-textured materials [7,16], with a strong affinity for water due to the presence of the abundant hydroxyl groups of cellulose. Numerous efforts have been made in order to turn these materials into water-resistant multifunctional systems, thus expanding their applicability in diverse technological fields [17,18,19,20]. To this end, studies have been conducted on the surface modification of cellulosic FP, which is commonly used for particle separation/filtration processes [9], in order to be utilized as an oil–water separator [5,6,7,8,9,10,11]. However, the majority of the proposed treatments demand complex preparation processes, some of the treatments have a high cost, the materials utilized limit the recyclability of the final system, and in some cases is difficult to scale up the fabrication process [6,7]. Nevertheless, utilization of cellulose fibers in large scale water-oil separation applications presents great advantages due to their abundance, low cost, biodegradability and excellent thermal and chemical stability. Therefore, the ideal cellulose substrate treatment should be of low cost and easily reproduced, the resulting material should be recyclable, and it should have high separation efficiency [6,21].

Herein, we describe a novel surface-treated FP for oil–water separation from non-stabilized emulsions prepared using commercially available hydrophobic paraffin wax (PFW) and Poly(dimethylsiloxane)-*b*-poly(ethylene oxide) (PDMS-*b*-PEO) diblock copolymer. The PFW treatment makes the FP hydrophobic and oleophilic. The subsequent application of PDMS-*b*-PEO offers the final desired properties to the FP, transforming it into a superhydrophilic and underwater superoleophobic porous material, able to separate oil from oil-in-water non-stabilized emulsions efficiently. In contrast to traditional separation processes, such as ultrafiltration membranes, where the applied driven-pressure for emulsion separation is hundreds of kPas, the proposed method demands much less energy in order to separate water from oil effectively. In fact, the separation process is gravity-driven [14,22], and allows to the water phase to pass through the FP, which at the same time completely repels the dispersed oil drops constricting them to remain on its surface, with separation efficiency of 99%. Key filter features including morphology, surface chemistry, wettability and mechanical properties show that PFW/PDMS-*b*-PEO treated FP can be successfully used for the separation of oil from oil-in-water, non-stabilized emulsions.

## 2. Materials and Methods

### 2.1. Materials

Toluene, methanol, paraffin wax (ASTM D 127, MP 70 °C–80 °C), light white mineral oil (density 0.84 g/mL at 25 °C), methylene blue, methyl orange, sudan blue dyes, and Whatman filter paper (grade 4, diameter 90 mm) were purchased from Sigma Aldrich, St. Louis, MO, USA. Other oils like olive oil, sunflower oil, and corn oil were purchased from a local market. Poly(dimethylsiloxane)-*b*-poly(ethylene oxide) (PDMS-*b*-PEO, *M*_w_: 600 g/mol, PDMS:PEO 25:75) was purchased from Polysciences Inc., Northampton, UK. All the above chemicals were used without any further purification. Deionized water was obtained from Milli-Q Advantage A10 ultrapure water purification system (Millipore, Billerica, MA, USA).

### 2.2. Fabrication of Superhydrophilic and Underwater Superoleophobic FP

**Figure 1:**
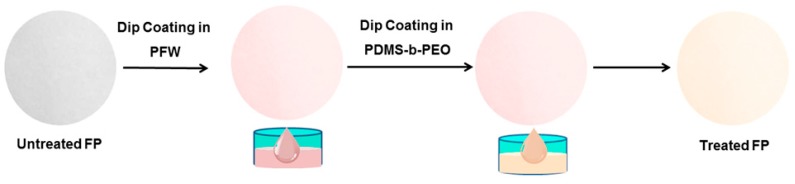
Schematic representation showing the fabrication steps of PFW/PDMS-*b*-PEO-treated FP.

PFW grains (average particle size 2 mm) were dispersed in toluene (5% and 10%, *w*/*v*) and the solutions were heated to 100 °C until clear solutions were obtained. The FP was then immersed in the warm (about 75 °C–80 °C) PFW solution for 30 s, removed and dried under an aspirated laboratory hood for about 1 h first and then in an air oven at 60 °C for 4 h. The PFW treated FPs were then dipped in PDMS-*b*-PEO/methanol solutions (0.5%, 1.0%, 1.5%, and 2.0%, *w*/*v*) for 30 s, removed and dried in the same way as in the PFW treatment. A schematic of the fabrication process and the identification names of the treated FP at different treatment conditions are given in Figure 1 and Table S1 (Supplementary Materials).

### 2.3. Characterization Studies

#### 2.3.1. Scanning Electron Microscopy (SEM)

The surface morphology of the FPs was investigated by scanning electron microscopy (SEM), using a variable pressure Jeol JSM-6490LA (JEOL, Tokyo, Japan) microscope equipped with a tungsten (W) thermionic electron source working in high vacuum mode, with an acceleration voltage of 10 kV. The test specimens (0.5 cm × 0.5 cm *c.a.*) were mounted on aluminum stubs with carbon tape. Before starting the measurements the samples were sputter coated with 10 nm of gold using a high resolution sputter coater (Cressington 208 HR, Cressington Scientific Instruments Ltd., Watford, UK).

#### 2.3.2. Porosity Measurements

The FP porosity was measured by completely filling the pores of treated and untreated paper samples of known weight and dimensions with silicon oil (Sigma Aldrich, viscosity 500 Cst, density 0.97 g/mL, and surface tension 21 dynes/cm), as stated in [23]. For this, 90 mm diameter FPs (untreated and treated) were first weighed in a precision balance and then each one was immersed in 10 mL silicon oil for 30 min. After immersion in silicon oil, the FP was gently pressed between two tissue papers in order to remove the excess surface oil. The porosity of the FP was then determined using Equation (1).

(1) $$Porosity\;\left(\%\right)=\frac{(C_{f}-C_{i})\rho }{C_{f}}\times 100$$

Here, *C*_i_ is the weight of FP before silicon oil saturation, *C*_f_ is the weight of the silicon oil saturated FP, and ρ is the density of silicon oil.

#### 2.3.3. Fourier Transform Infrared Spectroscopy-Attenuated Total Reflection (FTIR-ATR)

FTIR-ATR spectra of the FPs (treated and untreated) were recorded by Brucker V70 FTIR instrument (Bruker Analytik GmbH, Rheinstetten, Germany) equipped with an ATR unit (zinc selenide–ZnSe crystal). Scanning was run from 4000 to 400 cm^−1^ with 64 repetitive scans averaged for each spectrum and scan resolution was 4 cm^−1^. Spectra results obtained for the treated FP were compared with that of the untreated FP.

#### 2.3.4. Mechanical Characterization

The mechanical properties of the FPs (treated and untreated) were measured according to ASTM D 638-02a standard test method for tensile properties of plastics, at ambient laboratory conditions (*i.e.*, 21 °C and 50% RH). The dog bone-shaped specimens of the samples were characterized using a 5 kN Instron dual column tabletop universal testing system with 0.5 kN preload (T.A. Instruments, Instron model 3365, Norwood, MA, USA) and a crosshead rate of 5 mm/min. The tensile strength, percentage elongation at break, and Young’s modulus were calculated for each specimen. At least five samples were tested for each type of treatment and the results were averaged to obtain a mean value.

#### 2.3.5. Wettability and Contact Angle Measurements

Static and dynamic water contact angle measurements were carried out on rectangular strip samples using a contact angle goniometer DataPhysics OCAH 200 (Kruss GmbH, Hamburg, Germany) equipped with a CCD camera and image processing software, operating under laboratory conditions. Water droplets of volume 5 and 2 µL were placed on the surface of the samples and the static contact angle was measured within 45 s. The dynamic contact angle was recorded in a time range between 0.0 s (immediately after the droplet deposition) and 3.5 s. Up to ten measurements were conducted at random locations for each treated sample and the results were averaged to obtain a mean value. For underwater static and dynamic oil contact angle testing, the untreated and treated FPs were firmly placed at the bottom of a square optical glass cell (GC 10, no. 6000017, Data Physics, Kruss GmbH, Hamburg, Germany) with double-sided tape. The glass cell was filled with 6 mL of millipore water in order to completely cover the sample. A 12 µL dichloromethane (DCM) droplet was generated underwater on the paper sample and the contact angle was measured. At least 5 measurements at random positions were conducted and an average value was calculated.

#### 2.3.6. Separation of Oil from Oil-in-Water Non-Stabilized Emulsions

**Figure 2:**
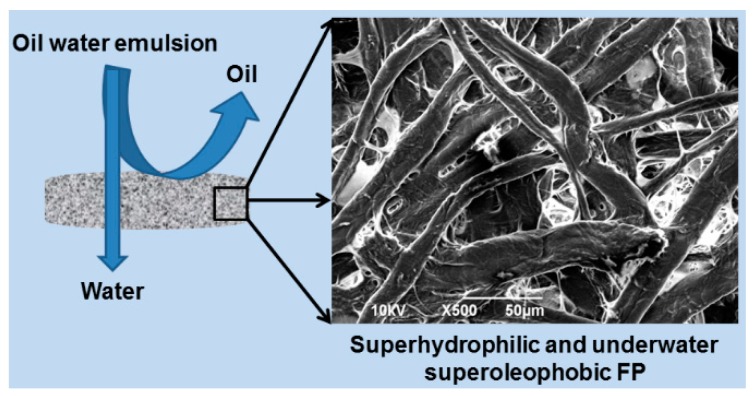
Schematic representation of the separation of oil and water from a non-stabilized oil-in-water emulsion.

Separation of oil and water was carried out as per the setup illustrated in the Figure 2. Two types of oil-in-water emulsions were studied: the first consisting of 80% water and 20% oil, (0.01 wt % of methylene blue 59 dye was used for water coloring, for better visualization and distinction of the two liquids), and the second consisting of 90% water and 10% oil. The oil-in-water emulsion was prepared with a vortex mixer (Heidolph multi reax, Schwabach, Germany) at 500 rpm for 10 min and then the emulsion was poured onto the FP through a glass tube. It must be noted that no surfactants or emulsion stabilizers were used during sonic treatment of the oil–water mixtures. The resultant non-stabilized emulsions separation process during the gravity driven filtration process is depicted in Figure 2.

In all cases, the separation process is gravity driven. After the separation processes, the oil and water recovery (%) are determined to be the volume ratio of the separated over the initial oil or water, respectively, as shown in Equations (2) and (3).

(2) $$Water\;Recovery\;\left(\%\right)=\frac{V_{collected\;water}}{V_{initial\;water}}\times 100$$

(3) $$Oil\;Recovery\;\left(\%\right)=\frac{V_{collected\;oil\;}}{V_{initial\;oil}}\times 100$$

From Table S2 (Supplementary Materials), it can be seen that according to the preliminary results for a 10 mL emulsion (20% of oil-in-water), the treatment of the FP with 10% *w*/*v* PFW and 1.0% *w*/*v* PDMS-*b*-PEO in methanol exhibited better separation performance. Based on this, the untreated, 10% PFW, 1.0% PDMS-*b*-PEO and 10% PFW and subsequently 1.0% PDMS-*b*-PEO treated FP results are presented in this manuscript.

#### 2.3.7. UV-vis Measurements

In the separation system, the concentration of the mineral oil after filtration and the separation efficiency were estimated using a Varian CARY 300 Scan UV-visible spectrophotometer (Agilent Technologies, Santa Clara, CA, USA) according to a standard procedure [8,24]. Initially the absorption spectra of 2 mL of different concentrations (0.0–30.0 mg/L) of oil solutions were recorded after diluting the mineral oil in hexane. The intensity of the absorbance peak at 272 nm for each oil concentration was then plotted as shown in Figure S1 (Supplementary Materials) and the equation which gives the dependence of the intensity to the oil concentration was defined by a linear fit. For these measurements the baseline correction of hexane was used. Subsequently the absorption spectra of 2 mL of filtered oil, water and original (pristine) oil were recorded, and the concentration of oil was calculated by the intensity of the peak and the above mentioned linear fit in Figure S1 (Supplementary Materials). At least three repetitions were made for the determination of oil concentration in each sample.

## 3. Results and Discussion

Two diverse polymeric layers were applied on the FP, namely PFW and PDMS-*b*-PEO, by a two-step dip adsorption process forming a functional coating on the surface of each individual fiber. PFW is a white, odorless, water-insoluble long chain branch hydrocarbon molecule which contains 20 to 40 carbon atoms in its chain, and is used as a coating, water/moisture barrier, anti-caking and lubricating agent for paper and textiles [25,26,27,28]. PDMS-*b*-PEO is a nontoxic environmentally friendly hydrophilic semi crystalline diblock copolymer [29,30,31,32], which is used in order to modify the surface properties of polymers, e.g., transform a hydrophobic material into a hydrophilic one [33,34].

**Figure 3:**
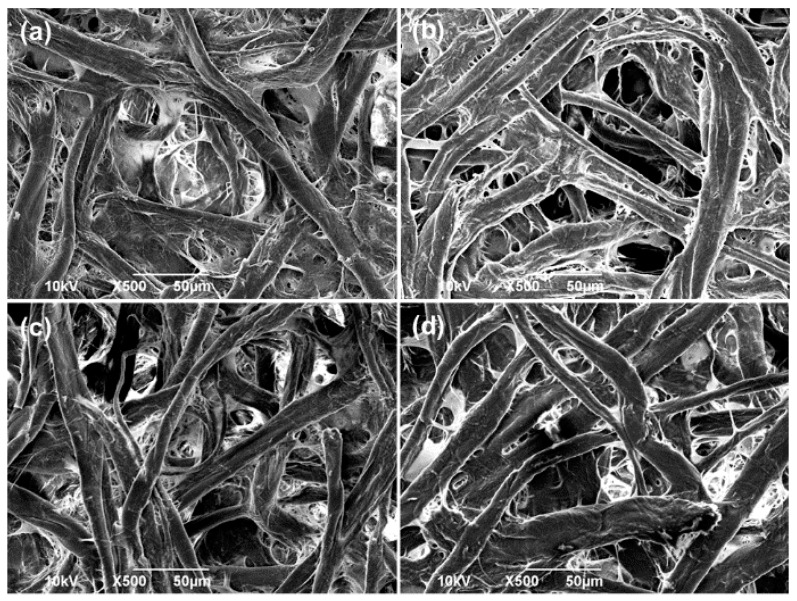
Scanning electron microscopy (SEM) surface analysis of (**a**) untreated, (**b**) PFW treated, (**c**) PDMS-*b*-PEO treated, and (**d**) PFW /PDMS-*b*-PEO treated FP samples.

Figure 3 shows the scanning electron microscope images (SEM) of the original (untreated) and the modified FP coated with PFW, PDMS-*b*-PEO, and PFW/PDMS-*b*-PEO. The untreated paper shows the interconnecting network of cellulose fibers with diameters of a few tens of micrometers, as shown in Figure 3a. When the untreated FP is dipped in the PFW solution, the latter penetrates in the innermost volume of the FP and coats all the fibers, forming a layer of 1.0 ± 0.1 µm as the cross section TEM analysis revealed (Supplementary Materials, Figure S2a,b). This thin layer does not significantly affect the network porosity and the morphology of the fibers which are very similar before and after the treatment as proved by the SEM micrograph in Figure 3b. After PFW treatment, FPs were subsequently treated with the PDMS-*b*-PEO solutions by dip-coating, and the overall morphology and structure of the porous network remained practically unaltered (Figure 3c,d). In fact, according to the cross section TEM analysis (Supplementary Materials, Figure S2c) the thickness of the polymer coating remains the same, 1.0 ± 0.1µm. The use of higher PFW concentrations did not show the same effect on the coating of the fibers since the polymer was forming a homogeneous compact film on the surface of the FP. On the other hand, higher PDMS-*b*-PEO quantities did not change the morphology of the treated paper.

The porosity study of the untreated and treated FPs shows that the applied PFW layer causes a slight decrease in the porosity of the untreated sample. In fact, as shown in the Supplementary Materials (Table S3), the mean porosity of the FP (53%), is decreased to 43% after the PFW treatment. The subsequent application of the PDMS-*b*-PEO layer does not significantly alter the porosity of the PFW coated FP (41% for the PFW/PDMS-*b*-PEO). Similarly, the sole PDMS-*b*-PEO treatment of the cellulose fibers does not have significant effect on the porosity (53.5%) of the untreated sample. This indicates that the PFW treatment forms a thick solid layer on the surface of each individual fiber whereas the PDMS-*b*-PEO layer is much thinner.

**Figure 4:**
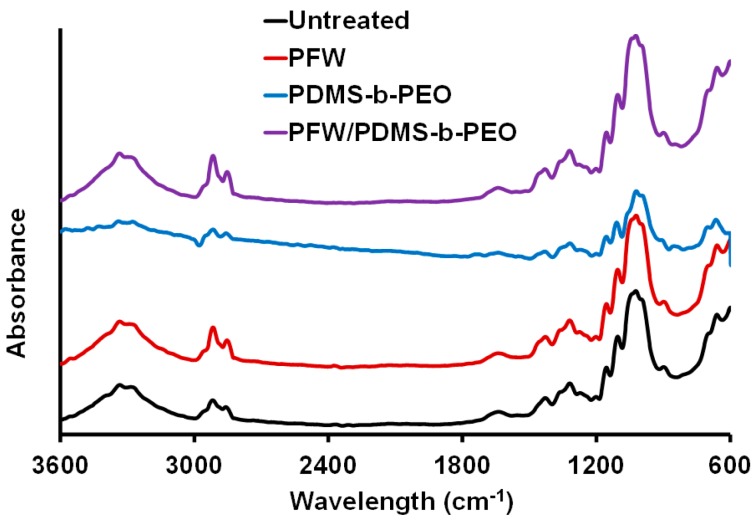
FTIR-ATR spectra of untreated FP, PFW, PDMS-*b*-PEO, and PFW/PDMS-*b*-PEO treated FP samples.

Figure 4 shows the FTIR-ATR spectra of untreated and treated FPs. Typical FTIR-ATR spectra of cellulose show a broad, strong absorption peak at about 3331 cm^−1^ due to the O–H stretching vibrations arising from abundant free hydroxyl and hydrogen-bonded hydroxyl groups, and the O–H bending of the adsorbed water at 1632 cm^−1^. The vibrations at 2918, 2851, and 1310 cm^−1^ are attributed to C–H stretching, bending and rocking, respectively. The absorption band at 1427 cm^−1^ is assigned to the C–H scissoring motion of cellulose. Finally, the signals of anti-symmetrical bridge C–O stretching and C–O–C stretching vibrations appear at 1157 and 1030 cm^−1^ of cellulose [35,36].

In case of PFW and PFW/PDMS-*b*-PEO treated filters all the absorption bands are present almost in the same position as in the spectra of the pristine cellulose FP. As shown in Figure 4, the strong and broad band around 3331 cm^−1^ that corresponds to the –OH stretching vibrations of hydroxyl groups, is practically at the same position for the untreated, PFW, and PFW/PDMS-*b*-PEO -treated cellulose. The characteristic absorption bands of carbon chains (C–H) for cellulose are at 2851 and 2918 cm^−1^. These bands are also at the same position in case of PFW and PFW/PDMS-*b*-PEO treatment. Therefore, there are no specific differences between treated and untreated FPs indicating that the PFW and PDMS-*b*-PEO layers are bonded to the cellulosic FP only through strong physical interactions.

**Figure 5:**
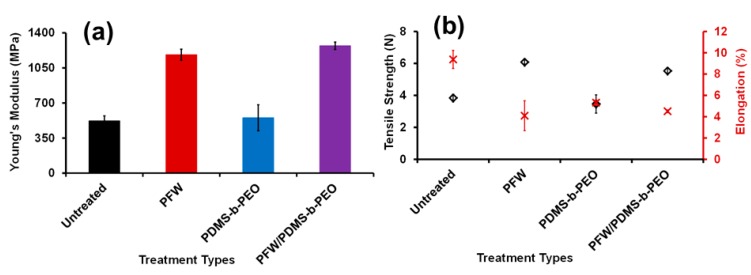
Young’s modulus (**a**), and tensile strength, and elongation percentage (**b**) of untreated, PFW, PDMS-*b*-PEO and PFW/PDMS-*b*-PEO -treated FP samples.

The mechanical strength of the pristine FP, PFW, PDMS-*b*-PEO, and PFW/PDMS-*b*-PEO -treated FPs was studied, and the results of Young’s modulus, tensile strength, and percentage elongation at break are shown in Figure 5. The elastic modulus of the FPs after being treated with PFW increases from 521.3 to 1.2 GPa, while their tensile strength increases from 3.8 to 6.1 N (Figure 5b). At the same time, the elongation at break decreases from 9.4% to 4.1%. We assume that the strong adhesion of the PFW on the cellulose fibers can induce physical cross-linking of the fibers and PFW, which turns the FP into a stronger material [17]. On the other hand, the PDMS-*b*-PEO treatment does not seem to have any influence to the mechanical properties of the FPs both in the untreated and PFW treated samples. In fact, after treatment of the PFW treated filters with the PDMS-*b*-PEO polymer, the excellent mechanical properties obtained due to the PFW are still maintained.

**Figure 6:**
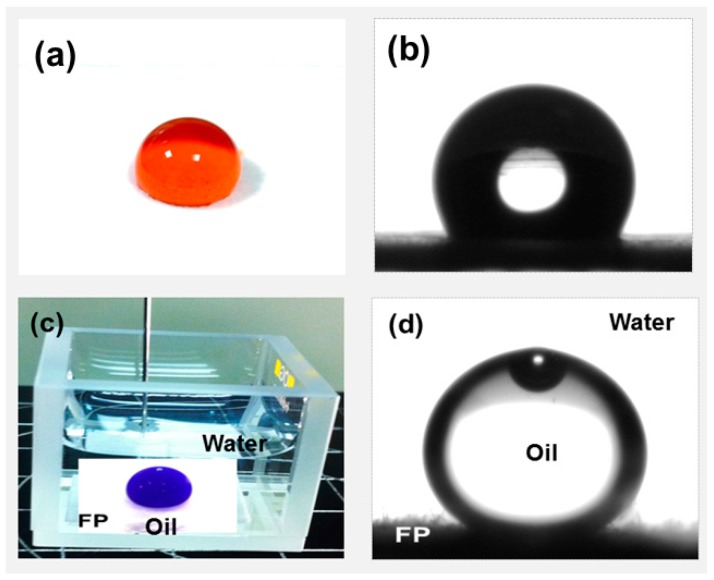
(**a**) Water droplet on PFW-treated FP. Methyl orange dye (0.01 wt %) is used to color the water, (**b**) water contact angle (125.8° ± 2.3°) of PFW-treated FP, (**c**) underwater DCM droplet on PFW/PDMS-*b*-PEO-treated FPs. Sudan blue dye (0.01%) is used to color the DCM, and (**d**) underwater DCM contact angle (148.7° ± 6.4°) of PFW/PDMS-*b*-PEO-treated FPs.

Water and oil (dichloromethane–DCM) static and dynamic contact angle tests in air and underwater on the untreated, PFW, PDMS-*b*-PEO, and PFW/PDMS-*b*-PEO treated FPs were carried out to investigate their wettability properties (Table 1, Figure 6 and Figure S3 Supplementary Materials). The untreated FP is rapidly wetted by water (after 80 ms) while the oil droplets are absorbed in less than few milliseconds (time below the instrument’s resolution), due to the abundant hydroxyl groups and cavities in its structure. In case of the PFW treatment, a hydrophobic coating is formed around each cellulose fiber, preventing water from wetting and penetrating. As shown in Table 1, Figure 6a and in Supplementary Materials Figures S3 and S4, when a water droplet is placed on the PFW-treated FP surface, it steadily remains on its surface, forming a water contact angle of 125.8° ± 2.3°. Conversely, the oil droplet wets the substrate immediately (faster than the instrument’s resolution). It should be noticed that lower amounts of PFW for the FP treatment were not sufficient to homogeneously coat the cellulose fibers, and therefore the water was slowly absorbed by the paper.

The subsequent PDMS-*b*-PEO treatment of the FP/PFW sample causes a remarkable change in the wetting properties, transforming the treated FP into a hydrophilic/oleophilic material with the water droplet being completely absorbed after 3.5 s, while the oil is absorbed immediately after the deposition (Figures S3 and S4 Supplementary Materials). In particular after dipping the PFW-treated FP into the PDMS-*b*-PEO solution, the PDMS block interacts with the PFW layer due to van der Waals forces and hydrophobic interactions, while the PEO, as a hydrophilic block, is exposed to the surface [31,32]. Therefore, the FP’s surface composition and properties depend on the fraction of the exposed PEO hydrophilic component. Higher amounts of PDMS-*b*-PEO for the treatment did not show any further improvement, while lower amounts were not enough so as to coat all the fibers and therefore the hydrophilicity of the FPs was reduced. The effectiveness of the PDMS-*b*-PEO component to hydrophilize surfaces is also demonstrated for the pure FPs, with the paper becoming even more hydrophilic and thus increasing the absorption speed from 80 to 60 ms, possibly due to the interaction of the copolymer with the hydrophobic components of the surface of the cellulose fibers. In the case of underwater oil contact angles, untreated, PFW, and only PDMS-*b*-PEO treated FPs are oleophobic with a stable oil contact angle over time of 134.7° ± 3.2°, 140.0° ± 1.6°, 142.4° ± 0.1° respectively. Surprisingly, when an oil droplet is placed on the surface of the PFW/PDMS-*b*-PEO FP underwater, it forms a static contact angle of 148.7° ± 6.40°, Table 1, and Figure 6d. In fact, due to the hydrophilicity of the treated FP, a water cushion between the oil droplet and the cellulose surface is formed underwater, with trapped water molecules within the FP network. This effect establishes the necessary conditions for the underwater superoleophobicity due to the repulsive interaction between polar (water) and non-polar (oil) molecules and the differences of their surface tension [1,14,15]. Underwater oleophobic properties are also observed when the oil droplet is placed on the wet PFW/PDMS-*b*-PEO FP surface in air as shown in Figure S4 (Supplementary Materials). On this wetted FP the oil droplet was not absorbed. Conversely, when the same experiment is repeated on the untreated, PFW and PDMS-*b*-PEO treated wet surface in air, the oil is absorbed within 1 min (see Figure S5 Supplementary Materials).

**Figure 7:**
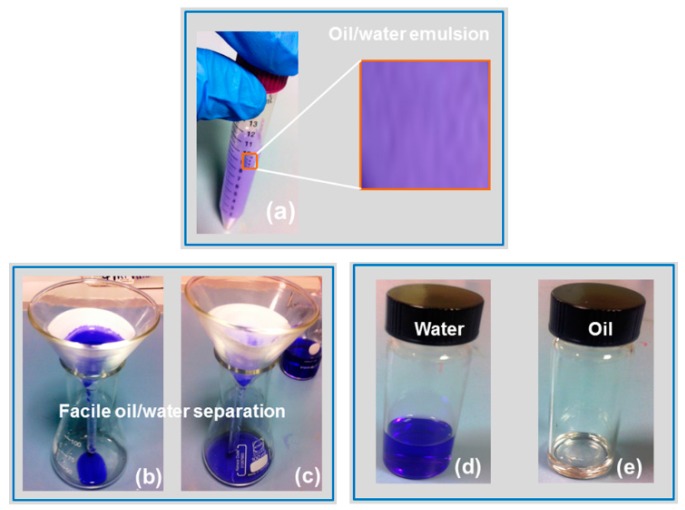
Different steps of oil–water separation experiments on 10 mL of oil-in-water non-stabilized emulsion. (**a**) Oil/water emulsion. Inset shows zoomed view for oil/water emulsion. Here, water is colored with methylene blue dye, in order to be distinguished from oil; (**b**,**c**) Separated water finishes at the bottom of the flask and separated oil remains on the filter paper; (**d**,**e**) Water and oil stored separately after filtration.

The underwater superoleophobic properties of the PFW/PDMS-*b*-PEO FP make it a promising candidate for oil–water separation applications. As a proof of concept, the oil-in-water emulsion separation is performed and sequentially shown in Figure 7. Ten milliliters of non-stabilized oil-in-water emulsion (80% water, and 20% oil) was poured onto the differently treated FPs. The untreated, PFW and PDMS-*b*-PEO -treated FPs were not able to completely separate the oil from the emulsion, as oil was completely adsorbed on the surface of the samples and its traces were observed on the filtered water (Figure S6, Supplementary Materials). However, due to the superhydrophilic and the underwater superoleophobic nature of the PFW/PDMS-*b*-PEO-treated FP, water can easily wet and penetrate the treated sample while the oil is trapped on its surface. As the microscopy study of the emulsion before and after the separation process showed, the maximum size of the non-stabilized emulsion droplets is 60 µm, (see Supplementary Materials Figure S7) while the minimum droplet size that can be separated by this process is 1.2 µm.

Four different kinds of oils were successfully separated from their water emulsions through the same process. The oils that were used were mineral oil (density: 0.840 g/L), corn oil (density: 0.930 g/L), olive oil (density: 0.860 g/L), and sunflower oil (density: 0.920 g/L). No visible residual oil was observed in the collected water, even after filtration of 100 mL of oil-in-water emulsions. This indicates that the treated FP can separate different kinds of oil with different densities. Since the average filtration time of 10 mL oil-in-water emulsion is about 55 s, then *c.a.* separation rate of oil–water is 77 L·m^−2^·d^−1^ (77 liters per meter square of FP per day), with the driving force being only the weight of the 10mL emulsion. Emulsions with higher volumes e.g., 1000 mL were also tested and similar results were found in terms of separation efficiency and rate, indicating that the treated FP can effectively separate large volumes of emulsions (Figure S8, Supplementary Materials).

**Figure 8:**
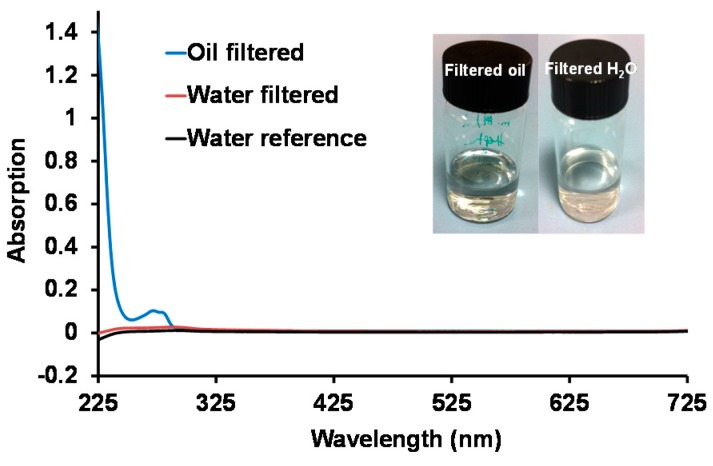
UV-vis absorption spectra of reference water (miliQ), filtered water and filtered oil.

To confirm the high separation efficiency, the potential presence of residual oil content in the filtered water was measured with UV-visible spectroscopy (Figure 8) [8]. As shown, the absorption curve of the filtered water is the same as the absorption curve for reference clean water, proving that there are hardly any oil contaminants present. On the contrary, a characteristic absorption band is observed in the filtered oil (mineral oil) absorption curve, with a peak at 272 nm [37,38] typical for the mineral oil. The absorption intensity of the peak is used in order to define the mineral oil concentration as discussed in the experimental section, and subsequently for the determination of the filtration efficiency of oil using Equation (4):

(4) $$Filtration\;Efficiency=\left(1-\frac{C_{f}}{C_{i}}\right)\times 100\%$$

where *C*_f_ is the concentration of oil in the filtered water and *C*_i_ is the initial concentration of oil in the non-stabilized oil–water emulsion. The filtration efficiency was calculated as 99.0% in accordance with the value calculated by the volumetric ratio.

## 4. Conclusions

In conclusion, we present a novel method to modify the surface properties of filter paper using polymeric solutions, rendering them superhydrophilic and underwater superoleophobic in a simple, straightforward and cost effective way. Various oils such as mineral oil, olive oil, and vegetable oil can be efficiently separated from their water emulsions based on the gravity-driven separation process. The filter paper becomes superhydrophilic and also exhibits underwater superoleophobic properties after PFW/PDMS-*b*-PEO treatment. The SEM and TEM analysis revealed that the morphology of the filter paper is not altered significantly while the mechanical properties of the system are improved. The superhydrophilic and underwater superoleophobic filter paper possesses excellent oil–water separation efficiency up to 99% and a rate of about 77 L·m^−2^·d^−1^. As such, the treatment presented here can be easily industrialized due to fact that both the substrate (cellulose) and polymers used are abundant and produced at industrial scales.

## Figures and Tables

**Table 1:** Water and oil (DCM) static contact angle (SCA) measurement of untreated, PFW, PDMS-*b*-PEO and PFW/PDMS-*b*-PEO treated FP in air and underwater.

Sample	SCA of water (°) air	SCA of DCM (°) air	SCA of DCM (°) underwater
Untreated	absorbed	absorbed	134.7 ± 3.2
PFW	125.8 ± 2.3	absorbed	140.0 ± 1.6
PDMS-*b*-PEO	absorbed	absorbed	142.4 ± 0.6
PFW/PDMS-*b*-PEO	absorbed	absorbed	148.7 ± 6.4

## Supplementary Materials

The supplementary materials can be found at www.mdpi.com/2073-4360/8/2/52/s1; Table S1. Filter paper different treatment conditions; Table S2. Water contact angle, water/oil recovery percentages, filtration time, and young’s modulus of different treated and untreated filter papers; Figure S1. Calibration curve of mineral oil; Figure S2. Cross-sectional TEM images of the untreated, and treated FPs; Table S3. Porosity of the untreated, and treated FPs; Figure S3. Dynamic water contact angle (DCA) of untreated and treated FPs; Figure S4. Wettability characterization of untreated and treated FPs in dry state; Figure S5. Underwater oil contact angles of untreated and treated FPs; Figure S6. Photographs of the filtered water after the emulsion separation using untreated and treated FPs; Figure S7. Microscopic analysis of the oil droplet size of non-stable emulsion droplets (20% oil-in-water) before and after filtration; Figure S8. Separation of large volume (1000 mL) of non-stabilized emulsions through PFW/PDMS-*b*-PEO treated FP.
