# Supplementary material for: Functionalized Cellulose Networks for Efficient Oil Removal from Oil–Water Emulsions
Source: Polymers (Basel). 2016 Feb 17;8(2):52. doi: 10.3390/polym8020052 (PMC6432539; doi:10.3390/polym8020052)
Supplement: Supplementary file 1 [file polymers-08-00052-s001.pdf]

Supplementary Materials: Functionalized Cellulose Networks for Efficient Oil Removal from Oil-Water Emulsions

Uttam C. Paul, Despina Fragouli, Ilker S. Bayer and Athanassia Athanassiou

Table S1. Filter paper different treatment conditions.

Samples	Paraffin wax (%, *w*/*v*)	PDMS-*b*-PEO (%, *w*/*v*)
Untreated	--	--
5% PFW	5%	--
10% PFW	10%	--
0.5% PDMS-*b*-PEO	--	0.5%
1.0% PDMS-*b*-PEO	--	1.0%
1.5% PDMS-*b*-PEO	--	1.5%
2.0% PDMS-*b*-PEO	--	2.0%
5% PFW/0.5% PDMS-*b*-PEO	5%	0.5%
5% PFW/1.0% PDMS-*b*-PEO	5%	1.0%
5% PFW/1.5% PDMS-*b*-PEO	5%	1.5%
5% PFW/2.0% PDMS-*b*-PEO	5%	2.0%
10% PFW/0.5% PDMS-*b*-PEO	10%	0.5%
10% PFW/1.0% PDMS-*b*-PEO	10%	1.0%
10% PFW/1.5% PDMS-*b*-PEO	10%	1.5%
10% PFW/2.0% PDMS-*b*-PEO	10%	2.0%

Table S2. Water contact angle, water / oil recovery percentages, filtration time, and young's modulus of different treated and untreated filter papers.

Sample	Water CA (°)	Water recovery (%)	Oil recovery (%)	Filtration time (s)	Young's Modulus (MPa)
Untreated	0.0	85.6	85.0	147	521.3 ± 50.1
5% PFW	125.8 ± 4.0	93.75	91.5	570	1389.2 ± 54.2
10% PFW	125.8 ± 2.8	97.5	92.0	546	1181.3 ± 55.9
0.5% PDMS-*b*-PEO	0.0	87.5	99.0	70	524.6 ± 90.2
1.0% PDMS-*b*-PEO	0.0	87.5	98.0	124	552.4 ± 129.5
1.5% PDMS-*b*-PEO	0.0	86.3	98.0	89	523.7 ± 125.2.
2.0% PDMS-*b*-PEO	0.0	85.0	97.5	59	661.9 ± 148.8
5% PFW/0.5% PDMS-*b*-PEO	49.3 ± 10.7	98.1	95.0	201	1037.5 ± 92.6
5% PFW/1.0% PDMS-*b*-PEO	9.3 ± 9.1	98.8	95.5	135	1238.6 ± 62.9
5% PFW/1.5% PDMS-*b*-PEO	0.0	91.3	97.0	67	937.8 ± 63.1
5% PFW/2.0% PDMS-*b*-PEO	0.0	87.5	98.0	75	843.3 ± 101.5
10% PFW/0.5% PDMS-*b*-PEO	60.1 ± 13.2	95.0	95.0	153	1221.8 ± 132.1
10% PFW/1.0% PDMS-*b*-PEO	0.0	93.8	99.0	55	1271.0 ± 37.0
10% PFW/1.5% PDMS-*b*-PEO	0.0	93.1	99.0	52	1030.8 ± 145.8
10% PFW/2.0% PDMS-*b*-PEO	0.0	88.8	99.0	51	864.1 ± 77.1

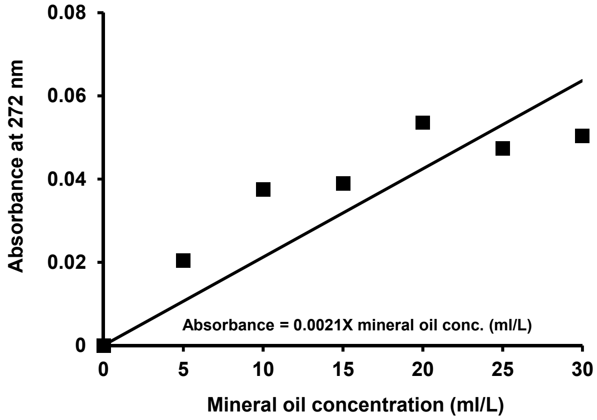

Figure S1. Calibration curve of mineral oil.

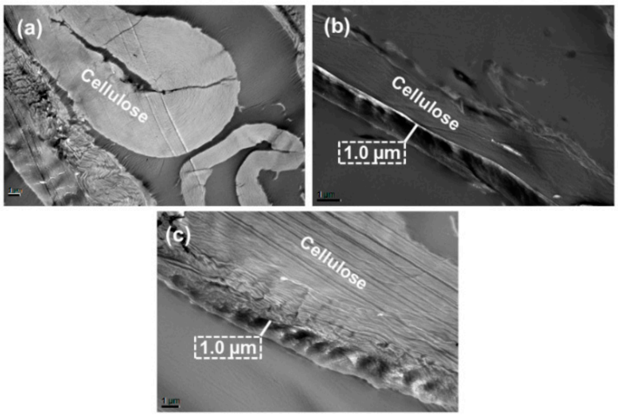

Figure S2. Cross-sectional TEM images of the (**a**) untreated, (**b**) PFW, and (**c**) PFW/PDMS-*b*-PEO treated FP. TEM images were collected with a JEOL JEM 1011 electron microscope, operating at an acceleration voltage of 100 kV, with a 11 Mp fiber optical charge-coupled device (CCD) camera (Gatan Orius SC-1000).

Table S3. Porosity of the untreated, PFW, PDMS-*b*-PEO, and PFW/PDMS-*b*-PEO treated FP.

Sample	Porosity
Untreated FP	52.9% ± 0.1%
10% PFW treated FP	42.8% ± 0.9%
1.0% PDMS-*b*-PEO treated FP	53.5% ± 0.2%
10% PFW/1.0%PDMS-*b*-PEO treated FP	40.8% ± 0.1%

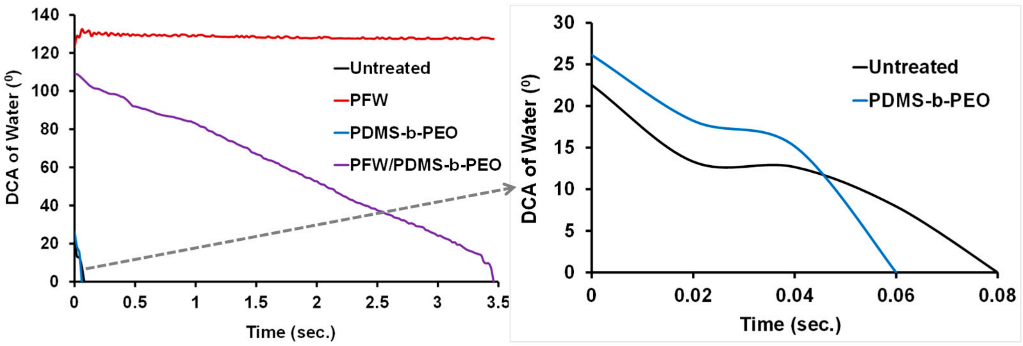

Figure S3. Dynamic water contact angle (DCA) of untreated, PFW, PDMS-*b*-PEO, and PFW/PDMS-*b*-PEO treated FP.

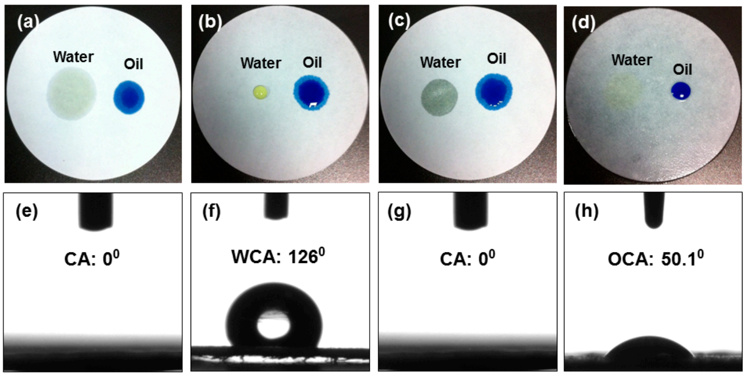

Figure S4. Wettability and contact angle (CA) of (**a**,**e**) untreated, (**b**,**f**) PFW treated FP, (**c**,**g**) PFW/PDMS-*b*-PEO treated FP in dry-solid state, and (**d**,**h**) water adsorbed PFW/PDMS-*b*-PEO treated FP. Here WCA means: water contact angle, OCA means: oil contact angle.

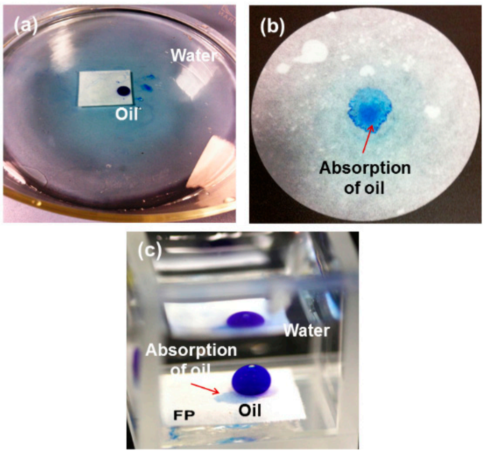

Figure S5. Underwater oleophobic properties of untreated and PFW treated FP. (**a**) Oil (DCM) droplet on untreated FP, (**b**) oil (DCM) droplet on wetted untreated FP, indicating the absorption of oil in wetted FP and (**c**) PFW treated FP, indicating the absorption of oil in underwater FP.

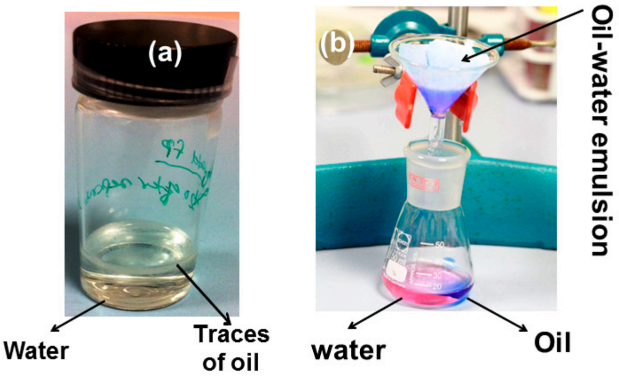

Figure S6. Photographs of the filtered water after the emulsion separation using (**a**) untreated and (**b**) PFW treated FP, indicating the traces of oil floating on water. In Figure (**b**), the water was colored with food grade red colorant and the oil with Sudan blue, for better visualization.

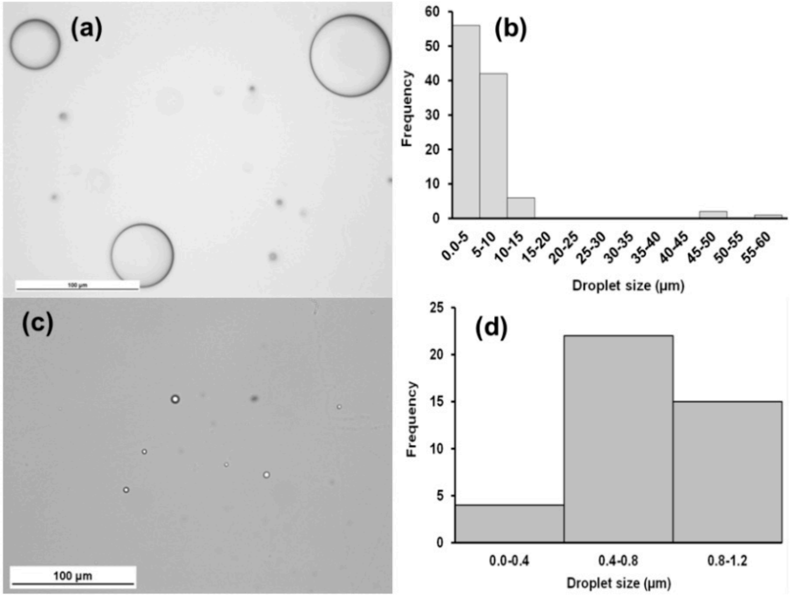

Figure S7. Microscopic analysis of the oil droplets size. (**a**) Microscopic image of non-stable emulsion droplets (20% oil in water), (**b**) size (μm) of the oil droplets of the non-stabilized emulsions before filtration, (**c**) microscopic image of the residual oil droplets in filtered water, and (**d**) size (μm) distribution of the oil droplets in filtered water. For the analysis of droplets size, the non-stabilized emulsions of 20% oil in water and the filtered water of PFW/PDMS-*b*-PEO were used at room temperature (21 °C). The droplet size distribution was obtained by placing a small drop of the non-stabilized emulsion and a small drop of filtered water on glass slides. The drop was then flattened by placing a smaller glass slide on top of the droplet. Thereafter, at least ten pictures of the non-stabilized emulsion droplets and filtered water were taken by a Nikon Eclipse 80i microscope equipped with NIS elements F software. The diameter of the droplets was then measured with ImageJ software. In order to get a reliable impression of the droplet sizes at least 100 droplets for non-stabilized emulsions and 40 droplets of filtered water were counted in the distribution population.

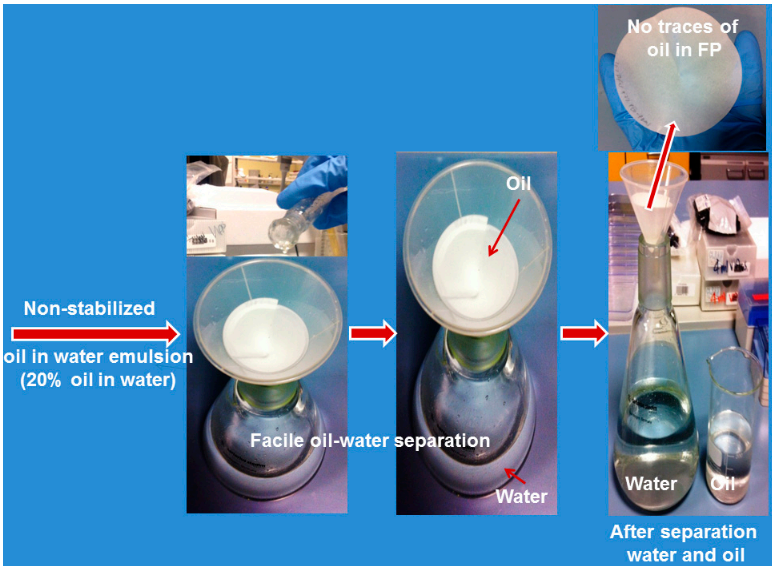

Figure S8. Separation of large volume (1000 mL) of non-stabilized emulsions through PFW/PDMS-*b*-PEO treated FP.

© 2016 by the authors; licensee MDPI, Basel, Switzerland. This article is an open access article distributed under the terms and conditions of the Creative Commons by Attribution (CC-BY) license (http://creativecommons.org/licenses/by/4.0/).
